# The Network of Services for COVID-19 Vaccination in Persons With Mental Disorders: The Italian Social Health System, Its Organization, and Bioethical Issues

**DOI:** 10.3389/fpubh.2022.870386

**Published:** 2022-06-20

**Authors:** Roberto Scendoni, Piergiorgio Fedeli, Mariano Cingolani

**Affiliations:** ^1^Department of Law, Institute of Legal Medicine, University of Macerata, Macerata, Italy; ^2^School of Law, Legal Medicine, University of Camerino, Camerino, Italy

**Keywords:** COVID-19 vaccination, mental disorders, healthcare system, health services, vaccine hesitancy, good practices, ethics

## Abstract

The adoption of restrictive measures aimed at curtailing the spread of SARS-CoV2 has had a harmful impact on socio-affective relationships, while limiting the scope of interventions and activities to promote social inclusion, with considerable negative repercussions for patients with mental disorders. Vaccination has been and will continue to be a valid tool to overcome the barriers of social isolation and to protect the health of this category of patients. In this paper we present an overview of the Italian network of social and healthcare services for COVID-19 vaccination among patients with mental disorders. Some aspects of medical ethics are discussed in order to share good practices for improving the health of this vulnerable group of people. We then consider the measures implemented by the health system in Italy to deal with the phenomenon of vaccine hesitancy before addressing the issue of autonomy and restricted access to vaccination points. Finally, we illustrate some of the perspectives already adopted by the Italian system, which may be useful to the global scientific community.

## Introduction

People with mental disorders, especially severe mental illness, have shown higher rates of COVID-19 morbidity and mortality (1); therefore, vaccination against COVID-19 should be prioritized for this vulnerable group, and this has already been assured in several countries (2, 3).

These patients may have difficulty in adhering to prevention measures (4) due to problems of understanding updated information or challenges in adapting their behavior according to the degree of risk.

The adoption of restrictive measures aimed at reducing the risk of contagion and circulation of SARS-CoV2 necessarily entailed an impoverishment of socio-affective relationships, limiting the scope of interventions and activities to promote social inclusion, negatively affecting the person's wellbeing and causing discomfort and a sense of isolation (5). Vaccination has proved to be an excellent tool for bypassing social isolation barriers. Although vaccination readiness varies widely across countries (6), some studies have shown that people with psychiatric disorders tend to be willing to receive vaccination and that vaccination rates in these populations can be increased by targeted prevention programs (7, 8). However, the threat of resistance or the patient's objective mental inability to accept the vaccination in an autonomous way are issues that should not be neglected. In this context, it is essential to improve the network of assistance services (hospital and territorial) that facilitate access to vaccines. Above all, efforts must be made to increase willingness to get vaccinated.

This paper describes the current organizational model covering Italy's network of vaccination services for patients with mental disorders.

## Legislative and Ethical Preconditions for Mental Health Assistance

As regards the issue of giving assistance to psychiatric patients in Italy, it is appropriate to highlight Law no. 180 of 1978 (9). The contents of this law were transferred and even more qualified in the law establishing the National Health System, again in 1978 (Law no. 833) (10), giving a unitary breath to all the matter that entered the general health system. This legislation certainly represented a scientific, cultural, and civil achievement, putting an end to the establishment of mental institutions and opening up new paths for the organization of a healthcare system, thereby creating the conditions for restoring full “citizenship” to psychiatric patients. The Italian model, sponsored by the World Health Organization (WHO), has influenced the mental health policies of many other countries aimed at replacing asylums with more effective and efficient forms of territorial assistance.

In this context, important issues of professional medical ethics emerge, which directly call into question the responsibilities of all organizations required to apply the aforementioned law. These aspects concern in particular:

- Proper training of social workers, psychologists, and medical specialists;
- The creation of rehabilitation structures providing different levels of protection;
- Greater attention to psychiatric assistance for minors, especially regarding the mental distress that occurs in adolescence;
- Greater intervention in prevention and early diagnosis;
- The care of seriously ill patients who refuse both medical and psychiatric treatment and are at risk of showing violent behavior;
- Public information and discussion to fight against prejudice toward those with mental illness.

WHO recognizes the essential role of mental health in a person's overall state of wellbeing and ability to interact with others. Evidence indicates that mental disorders, one of the main sources of suffering and disability in the world, are progressively increasing. Due to their complex etiopathogenesis involving other physiological systems, in addition to their chronicity and their effects on the nervous system, mental disorders require a multidisciplinary approach that supports clinical research into biological and psychosocial factors that contribute to vulnerability and the ability of an individual to cope (resilience) with such pathologies. To this end, the Istituto Superiore di Sanità (ISS) in the Italian system constantly promotes research and its application with particular attention to particularly critical phases such as the perinatal period, childhood, adolescence, and senescence.

Since 2018 a working group of the Reference Center for Behavioral Sciences and Mental Health, in line with WHO's Mental Health Action Plan 2013–2030 (11), has been carrying out a coordination action plan to strengthen the national network of psychiatric services, with the aim of disseminating and updating good practices to improve the health of people suffering from mental disorders, as well as their families and the general population (12).

In the psychiatric field, we are often faced with ethical issues: informed consent, the mandatory nature of treatment, professional secrecy, the equitable distribution of resources, and drug experimentation (13). Establishing a therapeutic relationship with the psychiatric patient is often complicated because doctor and patient do not share a similar socio-relational background and because there are barriers to a complete exchange of health information. The psychiatric patient may also encounter major difficulties on important issues: the risk of marginalization in the hospital and healthcare environment; little or no emotional and environmental support; and poor living conditions.

In Italy, for COVID-19 hospitalized patients with psychiatric comorbidities, there is a higher mortality rate than for other comorbidities (14). Furthermore, they are frail patients due to their reduced ability to independently manage their health interests, including (in this context) measures to contain the infection (distance, use of masks, hand washing, etc.). In some cases, these subjects frequent meeting places where the risk of contagion is much higher.

In this pandemic phase, now more than ever, there is a need to implement welfare and therapeutic measures for persons suffering from mental disorders, including minors (15), and to strengthen the network of assistance services for psychiatric patients. COVID-19 vaccination should not be used as a taxable act, but as a suitable tool to prevent forms of social isolation and aggravation of psychiatric illnesses.

## The National Network of Mental Health Services

In Italy, the network of mental health services (16) is structured as follows:

### Department of Mental Health (Dipartimento di Salute Mentale - DSM)

The Department of Mental Health (DSM) is the set of structures and services tasked with meeting demands linked to the care, assistance, and protection of mental health within the region defined by the local health authority (*Azienda Sanitaria Locale - ASL)*.

The DSM is equipped with the following services:

- Day care services: Mental Health Centers (*Centri di Salute Mentale*- CSMs)
- Semi-residential services: Day Centers (*Centri Diurni*- CDs)
- Residential services: residential structures (*Strutture Residenziali*- SRs) divided into therapeutic-rehabilitative and socio-rehabilitative residences
- Hospital services: the Psychiatric Diagnosis and Care Services (*Servizio Psichiatrico di Diagnosi e Cura*- SPDC) and day hospitals.

The scope of available assistance is completed by university clinics and private nursing homes.

### Mental Health Center (Centro di Salute Mentale)

The Mental Health Center (CSM) is the primary care provider for citizens with mental illness. It coordinates all interventions for the prevention, treatment, and rehabilitation of citizens with psychiatric pathologies within a given region.

The Center is headed by a multi-professional team consisting of at least one psychiatrist, a psychologist, a social worker, and a professional nurse.

The CSM ensures the following interventions:

- Psychiatric treatments and psychotherapies, social interventions, admission of patients to day centers or day hospital residential structures, and hospitalizations
- Psychiatric diagnosis and psychological interviews to identify appropriate therapeutic-rehabilitative and socio-rehabilitative programs
- Liaison with general practitioners (GPs) to provide psychiatric consultancy and to conduct collaborative therapeutic projects and training activities
- Specialist advice for “border” services (alcoholism, drug addiction, etc.), as well as for residential facilities for the elderly and disabled
- Filtering of hospitalizations and control of hospitalization in accredited private neuropsychiatric nursing homes, in order to ensure therapeutic continuity
- Assessment for the purpose of continuous improvement of the quality of the practices and procedures adopted
- Collaboration with voluntary associations, schools, and social cooperatives.

### Day Center (Centro Diurno)

The Day Center (CD) is a semi-residential structure with therapeutic-rehabilitative functions. It has its own team, possibly supplemented by operators from social cooperatives and voluntary organizations. It has suitable and adequately equipped premises. As part of personalized therapeutic-rehabilitation projects, this type of facility enables individuals to follow therapeutic strategies and to experiment and learn self-care skills in areas such as: the activities of daily living, the development of individual and group interpersonal relationships, and the search for employment. The CD can be managed by the DSM or by the private sector and social enterprises. In compliance with national standards for accreditation, relations with the DSM are regulated by specific agreements, which guarantee the continuity of taking charge in the care pathway.

### Residential Facilities (Strutture Residenziali)

Residential Facilities (SRs) are extra-hospital facilities where a key part of the therapeutic-rehabilitative and socio-rehabilitative program for persons suffering with psychiatric distress (sent by the CSM) is conducted; this is personalized and periodically verified. These structures aim to offer a network of relationships and emancipatory opportunities, within the framework of specific rehabilitation activities. The residential structures are differentiated according to the intensity of health care and have no more than 20 places. They are located in urbanized and easily accessible locations to prevent any form of isolation of the residents and to encourage social exchange. SRs can be created and managed by the DSM or by the private sector and social enterprises.

### Psychiatric Diagnosis and Treatment Service (Servizio Psichiatrico di Diagnosi e Cura)

A Psychiatric Diagnosis and Treatment Service (SPDC) is a hospital service where voluntary and compulsory psychiatric treatments are carried out in hospital conditions. This facility also provides consultancy activities for other hospital services. Located within a hospital (or university polyclinic), each SPDC contains up to 16 beds and is equipped with adequate space for shared activities. The total number of beds tends to be identified as one for every 10,000 inhabitants.

## COVID-19 Vaccination Targets for People with Severe Mental Health Disorders

Patients with psychiatric disorders should be considered a vulnerable group for COVID-19 infection and must be provided with early access to COVID-19 vaccination.

Promotion of COVID-19 vaccine uptake among patients with mental illnesses is an important public health priority at the moment, as emerging evidence clearly indicates that patients with psychiatric conditions are prone to higher rates of COVID-19 infection along with its complications (17). As several studies have demonstrated, rates of obesity, hypertension, diabetes, and lung disease (all conditions linked to severe outcomes of COVID-19 disease) in these people are higher than in the general population. Furthermore, there is a risk that the initial psychiatric condition of these patients may become aggravated after the disease if they recover from the COVID-19 infection (18).

Based on these premises, the Italian system has adopted a specific approach to the vaccination against COVID-19 among people with serious mental health problems. As proposed in several studies (19), the objectives are as follows:

- To reduce the risk of serious illnesses and deaths as a result of SARS-CoV-2 infection;
- To allow these individuals to protect their psychosocial well-being, maintain social relationships, and reactivate paths of social inclusion;
- To promote continuity in the care and operation of residential structures;
- To protect the physical health of family members who are indispensable in the care process and to support health professionals in medical-diagnostic procedures related to COVID-19.

Vaccination procedures are planned in accordance with the local health authorities who can anticipate and thoroughly verify the specific needs of patients and their caregivers. Indeed, ensuring quality approaches and summarize specific elements that lead to in-depth interventions to reach the best solution for the patient, is currently an aspect of continuous scientific study (20).

## Measures to Face the Phenomenon of Vaccine Hesitancy or Restricted Access to Vaccination Points

The success of a mass vaccination campaign is based not only on logistical and organizational efficiency and effectiveness, but also on the population's vaccine acceptance in order to achieve an adequate coverage rate. The speed with which anti-COVID-19 vaccines have been developed represents a great achievement for science, but it can generate anxiety, fear, and skepticism, fueling the phenomenon of so-called “vaccine hesitancy,” defined by WHO as the delay in acceptance or refusal of safe vaccines despite availability of vaccination services (21). It is a complex and contextualized phenomenon that varies over time and depends on the place and type of vaccine; demographic, socio-economic, and historical-cultural factors also come into play (22). The phenomenon is also related to misinformation, disinformation, and conspiracy theories which are spread in particular through social media. In addition, socio-economic and health inequalities, low levels of education, poor access to accurate information, and the lack of effective public health messages or targeted campaigns to tackle barriers to access are all aspects that can influence vaccine hesitancy among certain populations (23).

What has been done in Italy to address this phenomenon? First of all, a strong vaccination campaign was carried out as part of a plan drawn up by the Ministry of Health in collaboration with the Extraordinary Commissioner for Emergency, the Higher Institute of Health, and AIFA (the Italian Medicines Agency), and this was adopted with a specific decree in March 2021 (24). The aims of conducting a rapid campaign were to promote:

a) effective and timely distribution of vaccines;
b) constant monitoring of needs and supplies;
c) increased daily administration capacity, effective and timely distribution of vaccines, and higher numbers of daily administrations.

In particular, the campaign was structured around three operational lines:

- procurement and distribution
- needs monitoring
- increased levels of vaccine administration.

In addition to the communication campaigns, which targeted everyone, some Italian regions (e.g., Liguria, Veneto) directly included persons with serious mental illness in the category of “fragility” for which vaccination should be a priority.

In this context, many Italian health facilities have experimented with fully equipped mobile vaccination units, which visit residential health facilities and psychiatric centers scattered throughout a given region. This was inspired by initiatives to facilitate vaccine access in other countries, such as the United States (25), and it was recommended in the Decalogue for the Anti-Covid Vaccination Plan 19 by the Italian Society of Hygiene, Preventive Medicine and Public Health as a useful strategy for reaching marginal targets and communities (26).

The implementation of “social distancing” measures aimed at curtailing the spread of SARS-CoV2 has necessarily had a harmful impact on socio-affective relationships, as mentioned previously, causing a sense of isolation, especially among those who already feel socially excluded, and unmasking or amplifying mental disorders in psychiatric subjects (27).

Furthermore, in residential facilities it is challenging for these patients to adhere to preventive measures not only because of their clinical characteristics (perhaps partly non-collaborative or not self-sufficient) but also for reasons linked to the structural limitations of these communities (28).

In the maintenance of social relationships and in the reactivation of social inclusion paths in the context of various activities offered by community services, vaccination against SARS-CoV-2 represents an essential tool of integration by preventing and controlling COVID-19 in communities of people with severe mental health problems (29). For this reason, the figure of the caregiver (family and non-family) is fundamental. In Italy, the decision was taken to vaccinate mentally ill patients at the same time as their caregiver, guardian, or support administrator. Sharing the vaccination has proved to be a key source of support for persons with mental disabilities, giving them a sense of security and reassurance that the act is necessary to protect their health.

The Italian government has allowed all regions of the country to support GPs in promoting vaccination among this category of patients, possibly assisted by nurses. In particular, the vaccination can be administered in various contexts:

- at the GP's surgery;
- at the patient's home
- at out-of-patient facilities.

In-home vaccination is currently reserved for patients for whom transport to the clinic or vaccination centers is contraindicated.

This operating mode is preceded by a telephone triage to verify the presence or absence of suspected COVID-19 symptoms, as well as the existence (or not) of temporary or absolute contraindications to vaccination.

Finally, the Italian Society of Psychiatry (SIP), in addition to underlining the importance of making the vaccination procedure available as quickly as possible to these subjects (to be considered as priority vaccinations), has taken steps to encourage adherence through the intervention of all disease care facilities, where staff have been asked to provide each patient with appropriate counseling, following the indications of recent literature concerning the topic (30).

## Conclusions

People with severe mental health problems have a high risk of contagion and mortality linked to COVID-19, and should therefore have priority access to vaccination.

In light of the above, the following recommendations are proposed for other countries, based on the conclusive indications of practices that have already been adopted by the Italian system:

- Maintain vaccination priority criteria that target patients with mental disorders in the continuing vaccination program;
- Implement measures for the improvement and implementation of social and health services, establishing national funds to allow for the recruitment of nurses, social workers, psychologists, and doctors (31);
- Create and constantly update guidelines or *ad hoc* protocols (with an “ethical” slant that takes into account the attitudes of this type of patient toward the vaccine), including adequate information on the benefits and risks of vaccination, while continuing to evaluate vaccine efficacy and safety and potential interactions with psychiatric drugs;
- Coordinate a coherent and constantly updated information flow, anticipating any critical issues and preparing personalized communications;
- Carry out an adequate assessment of the person's mental state, their decision-making capacity, and convictions regarding refusal or hesitation;
- Guarantee the presence of the caregiver or reference figures in the pre- and post-vaccination phase.

To overcome vaccine hesitancy, vaccination campaigns (32) should provide for the possibility of monitoring the adhesion of peripheral non-hospital micro-areas: only in this way can they be effective and avoid generating discrepancies and social difficulties even among patients with psychiatric disorders, who are already severely affected by the pandemic.

In the future it would be useful to develop a study in collaboration with psychiatric research units, considering specific indicators relating to the patient's personality (e.g., conscientiousness, agreeableness, extroversion, neuroticism, openness) (33) and a stratification of the various psychiatric pathologies. This would lead to the identification of different vaccination priority levels based on the type and severity of mental illness. In fact, the priority in vaccinating certain categories of psychiatric patients may not only be justified for individual wellbeing but also for the community, since during the acute phases of the disease these people may not be able to obey the rules of public health protection (in terms of masking, distance and hygiene) resulting in an exponential risk of catching the virus and spreading it (34).

Finally, independent vaccine safety studies are needed to strengthen vaccine confidence in patients with mental disorders (such studies have already been conducted for other categories of people) (35).

## Author Contributions

RS drafted the document and acquired the information. PF contributed to the substantial conception of the work. MC analyzed the regulatory information and reviewed it critically. All authors contributed to manuscript revision, read, and approved the submitted version.

## Conflict of Interest

The authors declare that the research was conducted in the absence of any commercial or financial relationships that could be construed as a potential conflict of interest.

## Publisher's Note

All claims expressed in this article are solely those of the authors and do not necessarily represent those of their affiliated organizations, or those of the publisher, the editors and the reviewers. Any product that may be evaluated in this article, or claim that may be made by its manufacturer, is not guaranteed or endorsed by the publisher.
